# The Effect of Processing Methods on Food Quality and Human Health: Latest Advances and Prospects

**DOI:** 10.3390/foods11040611

**Published:** 2022-02-21

**Authors:** Milan Houška, Filipa Vinagre Marques Silva

**Affiliations:** 1 Food Research Institute Prague, 102 00 Prague, Czech Republic; 2 LEAF, Linking Landscape, Environment, Agriculture and Food, Associated Laboratory TERRA, Instituto Superior de Agronomia, Universidade de Lisboa, Tapada da Ajuda, 1349-017 Lisboa, Portugal; fvsilva@isa.ulisboa.pt

This Special Issue is focused on the use of modern food processing technologies to retain the highest possible content of health-promoting compounds in raw foods. Additionally, these technologies enable the amount of salt and preservatives added to foods to be reduced. These additives are known to be prejudicial to human health, and are associated with a variety of health problems. This collection includes four reviews and six original research articles [1,2,3,4,5,6,7,8,9,10]. The review on high-pressure processing (HPP) technology is focused on fruit and vegetable products [1]. The effect of HPP on microorganisms and enzymes, and the historical evolution of the size of HPP machinery, are also documented. This review paper on HPP can also be used for educational purposes, as HPP is already a commercial, non-thermal technology used globally by the food and beverage industries [1].

Power ultrasound is another technology that can be used alone or to improve the efficiency of traditional food processes. Apples were sonicated before drying, which sped up the drying process and improved the product quality [2]. One other study presented results of the effect of ultrasound processing of tomato vinegar on its bioactive compounds. The following health-related quality parameters were investigated: cupric-reducing antioxidant capacity, and total flavonoid, total phenolic, total ascorbic acid and total lycopene contents [3]. 

Another review is focused on the potential application of non-thermal technologies to stabilize wines, as an alternative to the SO_2_ preservative, added to wine at different stages of wine production. The review showed minimal impact of pulsed electric fields (PEF), HPP and ultrasound on the quality of the wine [4]. A comparative study of the application of HPP, ultrasound and PEF for the pre-treatment of apples before air drying is included in this collection [5].

Sulforaphane exhibits a whole range of unique biological properties, and is present at 10 to 100 times higher levels in broccoli sprouts than in most mature plants. An interesting study demonstrated how to retain the sulforaphane content of broccoli sprouts during various processing steps (e.g., extraction) [6]. Another article showed the effect of the cultivation type and cooking method on the nutritional quality of Brazilian vegetables [7]. A review about strategies to reduce the salt content of a variety of foods, so that they become healthier, is also included in this collection [8]. The potential use of non-thermal technologies to preserve food and reduce sodium in foods is discussed. 

An optimization study of the extrusion process of whole black-grained wheat flour, to improve the physical properties and maximize the nutritional components, is published in this Special Issue [9]. A review paper focusing, once again, on extrusion cooking and how this process affects the phenolic content and other food components is included in this collection [10].
